# FeFET-Based Computing-in-Memory Unit Circuit and Its Application

**DOI:** 10.3390/nano15040319

**Published:** 2025-02-19

**Authors:** Xiaojing Zha, Hao Ye

**Affiliations:** 1 Faculty of Electrical Engineering and Computer Science, Ningbo University, Ningbo 315211, China; 2 College of Electrical and Electronic Engineering, Wenzhou University, Wenzhou 325000, China; yehao@wzu.edu.cn

**Keywords:** computing-in-memory, ferroelectric field-effect transistor, digital circuit design

## Abstract

With the increasing challenges facing silicon complementary metal oxide semiconductor (CMOS) technology, emerging non-volatile memory (NVM) has received extensive attention in overcoming the bottleneck. NVM and computing-in-memory (CiM) architecture are promising in reducing energy and time consumption in data-intensive computation. The HfO2-doped ferroelectric field-effect transistor (FeFET) is one of NVM and has been used in CiM digital circuit design. However, in the implementation of logical functions, different input forms, such as FeFET state and gate voltage, limit the logic cascade and restrict the rapid development of CiM digital circuits. To address this problem, this paper proposes a Vin–Vout CiM unit circuit with the built-in state of FeFET as a bridge. The proposed unit circuit unifies the form of logic inputs and describes the basic structure of FeFET to realize logic functions under the application of gate-source voltage. Based on the proposed unit circuit, basic logic gates are designed and used to realize CiM Full Adder (FA). The simulation results verify the feasibility of FeFET as the core of logic operations and prove the scalability of FeFET-based unit circuit, which is expected to develop more efficient CiM circuits.

## 1. Introduction

**Figure 1:**
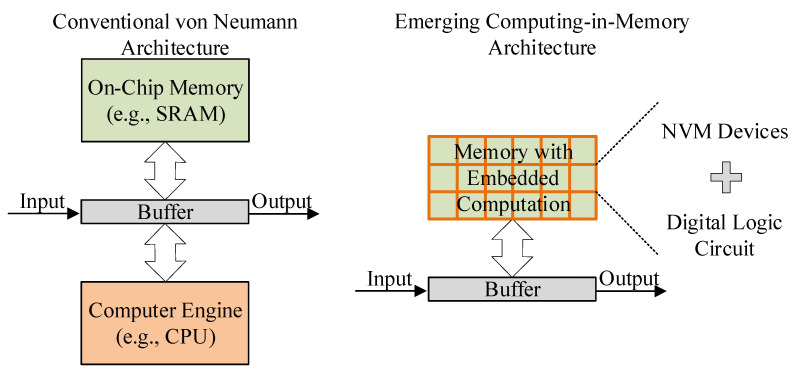
Schematic diagram comparing two computing architectures.

In recent years, emerging intelligence applications, such as the artificial intelligence of things (AIoT), robotics, and edge computing, have an increasing demand for chip computing power. However, the performance of such data-intensive computing has been limited by the physically separated logic and memory blocks in traditional von Neumann architecture, which is expected to be replaced by computing-in-memory (CiM) architecture. As shown in Figure 1, the CiM architecture combines logical operations and storage functions within memory units, significantly improving the processing and energy efficiency of data-intensive computing by reducing data movement between logic blocks and storage blocks. Toward such an efficient computing scheme, innovative nonvolatile memory (NVM) devices and corresponding digital logic circuits have been proposed to support CiM architecture.

From the perspective of materials and device structures, a number of recent efforts have attempted to design NVM devices, such as RRAM [1], PCAM [2], and MRAM [3]. Among a variety of emerging NVMs, HfO2-based ferroelectric field-effect transistors (FeFETs) have recently emerged as an appealing candidate for nonvolatile logic gates [4,5,6].

Based on the rich electrostatic physics and high CMOS compatibility, FeFETs have been widely used in special circuit fields such as memories [7], TCAMs [8,9], and CNNs (BCNNs) [10], etc. In terms of logic circuit design, some researchers have developed FeFET devices with rich computing functions through innovations in materials and structures [11,12,13]. Another group of researchers has developed the FeFET-based basic functional circuit, combining FeFET and CMOS devices from the perspective of circuit design. In circuit design, FeFETs are either regarded as storage elements to store logic inputs, implementing logic functions through the interconnection of circuit elements [14,15,16], or as switches, treating the FeFET switching state as internal input and the gate voltage signal as an external input, and performing operations on two different physical quantities [17,18,19]. However, the former only reflects the storage capacity of FeFET. Unless all logic inputs are stored in FeFET, the calculation results cannot be non-volatile. While the latter only reflects the computing capacity of FeFET based on the principle that the switching state can be regulated by the gate voltage. Due to the different physical forms of logic inputs, multiple power supplies and additional conversion circuits are required to realize the calculation cascades. The difficulty of calculation cascades and the volatility of calculation results limit the realization of complex logic functions. Therefore, existing circuit designs based on FeFET still face the challenges of high complexity and significant hardware overhead.

In order to effectively utilize the storage and computing power of FeFET and reduce the complexity of CiM circuit design, a FeFET-based unit circuit structure is proposed in this paper. The FeFET-based unit circuit adopts dynamic logic architecture, combined with a time-division gate-source voltage writing scheme, and performs built-in logic operations in FeFET according to the sequence of input voltage signals. The logic results can also be converted into voltage signals for output, which unifies the input and output forms. Based on the proposed unit circuit, basic logic gates are designed and used to implement CiM Full Adders (FAs). Since the unit circuit achieves voltage input–output when utilizing the computing power of FeFET, and the logic result can be stored in the FeFET, it shows the potential for optimizing circuit hardware resources and data backup overhead. Thus, a new idea for digital circuit design under the CiM architecture is provided.

## 2. Background

### 2.1. FeFET Device

**Figure 2:**
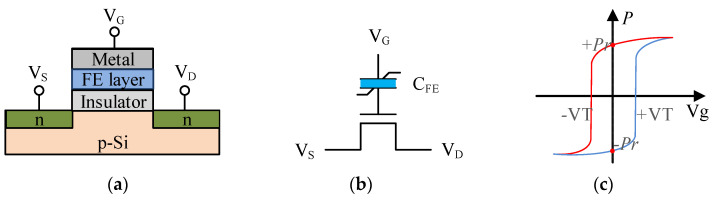
Structure and properties of FeFETs: (**a**) FeFET device structure; (**b**) the equivalent circuit is composed of a nonlinear ferroelectric capacitor in series with a MOSFET; (**c**) hysteresis loop and corresponding voltage operating range.

Figure 2a shows the widely studied metal-ferroelectric-insulator-semiconductor (MFIS) FeFET structure, which dopes HfO_2_ as ferroelectric layer in the gate stack of the transistor. The equivalent circuit is also shown in Figure 2b, where the ferroelectric capacitance (C_FE_) couples with the capacitance of the underlying MOSFET (C_MOS_). When the ratio of C_FE_/C_MOS_ is sufficiently low, the polarization state of the ferroelectric layer can be retained [20], resulting in a hysteresis characteristic curve, as shown in Figure 2c, which generates a storage window for the device.

The working principle of the FeFET is that the ferroelectric layer can exert a field effect on the channel through polarized charges, thereby controlling the threshold voltage of the transistor. For N-type FeFET, when a positive gate-source voltage Vgs > +VT is applied, the ferroelectric domain in the ferroelectric layer will flip to the positive polarization state, attracting electrons from the drain and source to the channel, resulting in an “ON” state corresponding to a low threshold voltage. Similarly, applying a negative gate-source voltage Vgs < −VT will flip the ferroelectric domain to the negative polarization state, increase the holes provided by the substrate, increase the threshold voltage, and the FeFET is in the “OFF” state. As shown in Figure 2c, this writing process has hysteresis characteristics, so when the gate-source voltage is not enough to flip the polarization state of the ferroelectric domain, the remnant polarization in the ferroelectric layer will maintain the device switching state, which enables NV functionalities.

Due to the inherent gain of the underlying MOSFET, the I_ON_/I_OFF_ ratio of FeFETs corresponding to the two switching states can be up to 10^6^ [14]. In addition, the three-terminal structure of FeFETs separates the state writing (by applying sufficient positive/negative Vgs) and state reading paths (via drain-source current). This is more compatible with MOSFET than other two-terminal NV devices (such as RRAM), providing more flexibility when considering application-driven circuit optimization.

### 2.2. FeFET Simulation Model and Writing Schemes

In order to achieve more efficient design and analysis of FeFET-based circuits, some works have combined new materials and new structural features to establish multi-domain mathematical models for FeFETs to accurately capture more device characteristics [21,22]. However, the binary logic operations discussed in this article do not rely on multi-domain dynamics, so a single-domain Landau–Khalatnikov (LK) model is used to analyze FeFET behavior [23].

**Figure 3:**
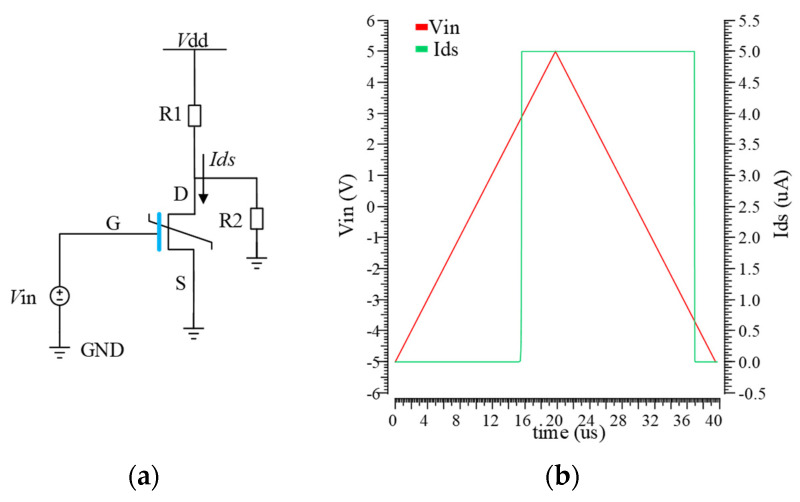
FeFET model simulation circuit and I-V curve: (**a**) the FeFET model simulation circuit. (**b**) the I-V characteristic of the adopted FeFET.

The LK equation describes the relationship between the applied electric field and the internal polarized charges within the gate. This equation is nonlinear:

(1) $$E=\alpha P+\beta P^{3}+\gamma P^{5}+\rho \frac{dP}{dt},$$

where *α*, *β*, and *γ* are the static coefficients and *ρ* is a kinetic coefficient associated with the ferroelectric material. In the rest of this paper, the calibrated 10nm model in [23] is used for experimental tests, with 0.25 kinetic coefficient *ρ*. The FeFET behavior is simulated by combining the self-consistent LK equation with the 180 nm technology model in the Cadence Virtuoso. The switching state of devices can be characterized by measuring the current-voltage (I–V) characteristic using the simulator Spectre, shown in Figure 3.

**Figure 4:**
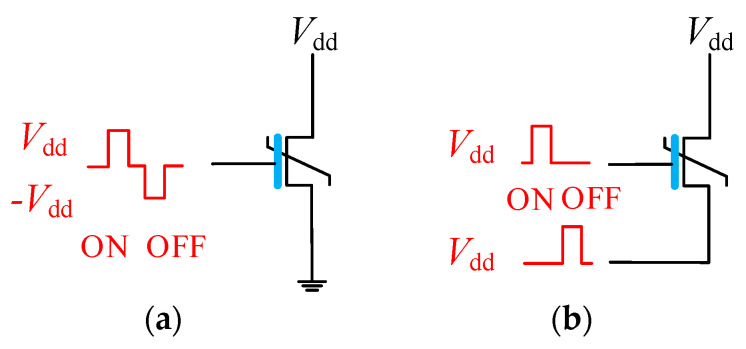
FeFET writing schemes: (**a**) Single gate positive and negative voltage input schemes. (**b**) gate-source writing scheme.

When designing FeFET-based memories, the two switching states of FeFET are generally corresponded to the logical value, so it is necessary to ensure stable state distinction [24]. The traditional solution is to apply positive and negative voltages to the gate for a long time to ensure the correct switching of FeFET, as shown in Figure 4a. However, the negative supply voltage not only increases the hardware cost but also limits the logic cascade. With the advancement of technology, the writing scheme of using gate-source voltage difference has gradually become the mainstream [14]. Figure 4b shows the gate-source writing scheme, where the source of FeFET is connected to the inverted gate voltage during writing to form a positive or negative voltage difference. Though the gate-source writing scheme has extra area overhead, it fully eliminates the need for an additional negative supply voltage and releases the FeFET source terminal as an independent input terminal, which is conducive to expanding the computing power of FeFET [25,26]. Therefore, the gate-source writing scheme is adopted in this paper.

### 2.3. FeFET Based Logic Operations

Several FeFET-based CiM designs have been proposed, ranging from application-specific designs to more general ones that focus on basic logics. In terms of dedicated circuit design, thanks to the three-terminal structure, FeFET-based TCAMs [8,9], multiplier [4], and adder [27], it achieves ultra-high density compared to other designs. In these works, FeFETs implement specific arithmetic functions by connecting CMOS transistors in series and parallel. However, in the above designs, FeFETs are only regarded as memory cells to store part of the logic inputs, lacking the embodiment of the computing potential of FeFETs, and the operation results still rely on additional memory cells to save.

Regarding general logic gate design, Breyer et al. first designed reconfigurable NAND and NOR logic gates based on the dual threshold characteristics of FeFET [28]. Then, complex logic functions such as XOR and XNOR are realized based on multi-device parallel connection and the top-gate and back-gate control strategies of FeFET [18]. Marchand et al. designed reconfigurable logic gates using complementary FeFETs and integrated these into a comprehensive computational architecture [17,19]. Huang et al. utilized the polarization state of ferroelectrics as a selection signal for a FeFET-based multiplexer (MUX) [29]. These studies have verified the feasibility of utilizing the polarization state of the ferroelectric layer or the switching state of FeFET as a logic input to participate in computing. Ding et al. heterogeneously integrated RRAM with FeFET and continued to use the switching state and gate voltage of FeFET as logic inputs to achieve a variety of logic operation functions with the help of different resistance states of RRAM [30]. Since the physical representations of logic inputs are different, and even the input and output logic quantities correspond to different voltage ranges: the logic variable represented by the switch state can be written in advance by applying a large voltage. The other logic variable is represented by a small voltage pulse. The above designs require multiple power pulses, and the output voltage also requires additional sensing amplifiers or conversion circuits to serve as the input of the next level of logic gates. Not only does it increase the complexity of the operation cascade but also limits the ability to describe complex logic functions.

Existing research has not comprehensively considered the storage and computing characteristics of FeFET, resulting in FeFET being used only as a storage or switching element, which limits the cascading and scalability of CiM circuits. To address this problem, this paper proposes a basic unit circuit from the perspective of unifying the input forms and the input and output voltage range and develops a general operation logic based on the proposed unit circuit, providing new design ideas for CiM scalable circuit design.

## 3. FeFET-Based CiM Circuit Design

### 3.1. FeFET-Based Unit Circuit

The dynamic logic (DL) design style has been exploited by many emerging NV devices. FeFETs, when replacing NMOS, are also suitable for building DL CiM circuits, and can ensure the consistency of input and output voltage ranges. However, the realization of different logic functions in previous designs depends on the series and parallel connection of FeFET and NMOS. The operation principle is basically the same as that of CMOS circuits, which ignored the computing advantage of FeFETs. The key difference between FeFET and MOS devices is that its switching state can participate in the operation as an internal input. If the switching state is only written through the gate voltage pulse, it will inevitably cause the input voltage to be inconsistent, which is not conducive to logic cascading. Therefore, a new module is needed to cooperate with the gate-source writing scheme to describe the connection between the internal input and the external voltage input.

**Figure 5:**
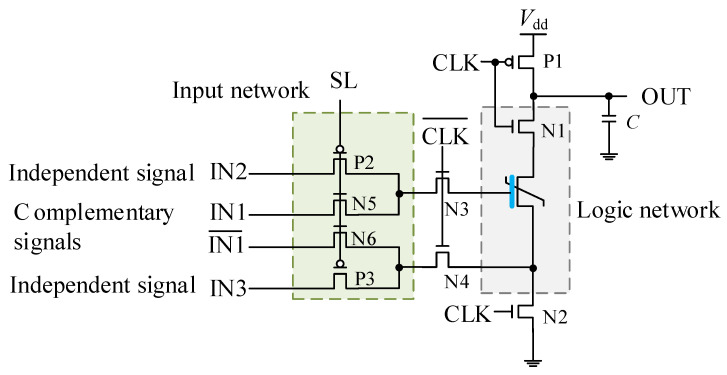
Schematic diagram of the FeFET-based unit circuit.

Figure 5 illustrates the basic unit structure evolved from the gate-source writing scheme. The difference between the proposed structure and the normal DL gate is that the logic network is formed by only one NMOS N1 and one FeFET in series. The NMOS N1 is used to isolate the influence of the pre-charge network on the FeFET, and the single FeFET device is used to implement the operation. In addition, an input network is added at the input end of the logic network to transmit multiple input signals to the gate-source of the FeFET in a time-division multiplexed manner. The logical functions that can be implemented by FeFET are determined by the timing voltage signal. The input network is connected to four input ports, of which IN1 and $\bar{\mathrm{IN}1}$ are a pair of complementary signals, and IN2 and IN3 are independent signals. The four input signals are controlled by the selection signal SL through NMOS N5/N6 and PMOS P2/P3, and the complementary signals will act on the gate and source of the FeFET, respectively, for setting the initial state of the FeFET. The two independent signals will be transmitted to the gate and source of the initialized FeFET, and the initial state of the FeFET will be maintained or switched according to the voltage difference in the independent signals. The final state of FeFET is uniquely determined by the input signals and reflects the operation process.

Performing a logic operation based on the basic unit circuit, following four steps:

(1) The output (*C*) is charged to Vdd: P1/N3/N4 turned on, N1 and N2 turned off.
(2) Complementary inputs are written to the FeFET: N5 and N6 turned on, P2 and P3 turned off.
(3) Independent inputs are fed to the FeFET: N5 and N6 turned off, P2 and P3 turned on.
(4) The state of FeFET is read out as a voltage signal: P1/N3/N4 turned off, N1 and N2 turned on.

**Figure 6:**
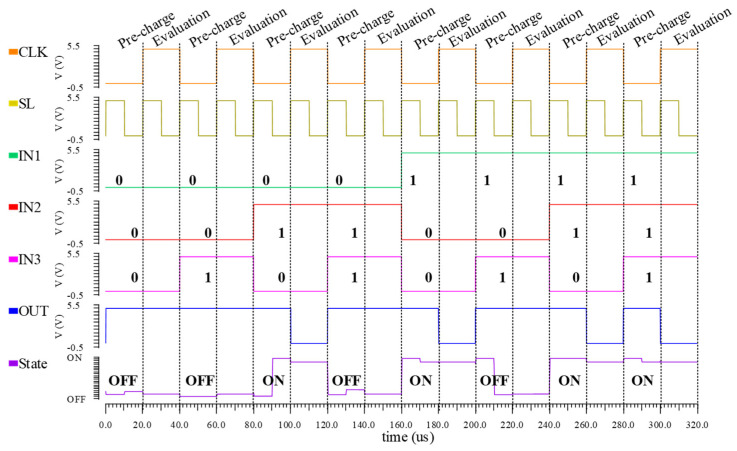
Sequential operation of FeFET-based unit circuit.

The sequential operation of implementing logic functions based on the unit circuit is shown in Figure 6, where the calculation occurs in the pre-charge phase (CLK is low, corresponding to steps 1–3), and the reading of the logic result occurs in the evaluation phase (CLK is high, corresponding to step 4). In the pre-charge stage, a pair of complementary voltages IN1 and $\bar{\mathrm{IN}1}$ first determine the state of the FeFET (SL is high, corresponding to step 2), and then the independent voltages IN2 and IN3 are calculated with the specific state (SL is low, corresponding to step 3). Since the complementary voltage is consistent with the independent voltage and the range of the output voltage, the logic operations performed on the unit circuit can unify the form and range of the logic inputs and overcome the overhead of additional conversion circuits. According to the simulation waveform, in the evaluation phase, if the FeFET is in the ON state, a conductive path will be established between Vdd and GND, otherwise, the output is maintained in the pre-charge phase equal to Vdd. The state of the FeFET is determined by the three inputs, so the unit circuit can realize the following functions:

(2) $$\mathrm{OUT}=\bar{\mathrm{CLK}}+\mathrm{CLK}•(\bar{\mathrm{IN}1}•\bar{\mathrm{IN}2}+\bar{\mathrm{IN}2}•\mathrm{IN}3+\bar{\mathrm{IN}1}•\mathrm{IN}3).$$

In addition, the logic network only contains FeFETs, and the calculation between the input voltages is carried out inside the FeFET device. The calculation result is presented as the non-volatile state of the FeFET, which can be read as a voltage in the evaluation stage. The amplitude of the output voltage is consistent with input voltages, and it can drive other unit circuits. Therefore, the unit circuit can simultaneously exhibit the computing and storage characteristics of FeFET.

### 3.2. Logic Gate Based on Unit Circuit

**Figure 7:**
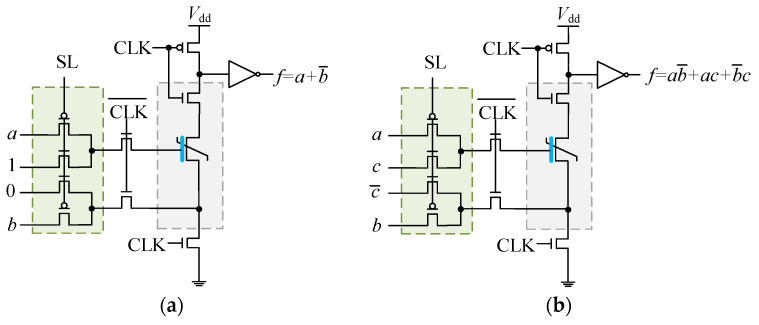
Schematic of logic gates based on the unit circuit: (**a**) IMP gate; (**b**) Built-in majority gate.

In order to ensure the complete non-volatility of the calculation results, the logic network of the unit circuit can only be composed of non-volatile devices. Therefore, the change in different logic functions is completely realized by adjusting the input signals and the series or parallel connection of FeFETs in the logic network. Let *a*, *b*, *c*, … represent binary signals, whose values “1” or “0” indicate the high or low voltage signals. In the unit circuit, the input network connects three independent inputs (one of which corresponds to the pair of complementary signals), and the output is connected to an inverter for shaping. The constant logic operation and variable logic operation based on them are shown in Figure 7.

**Figure 8:**
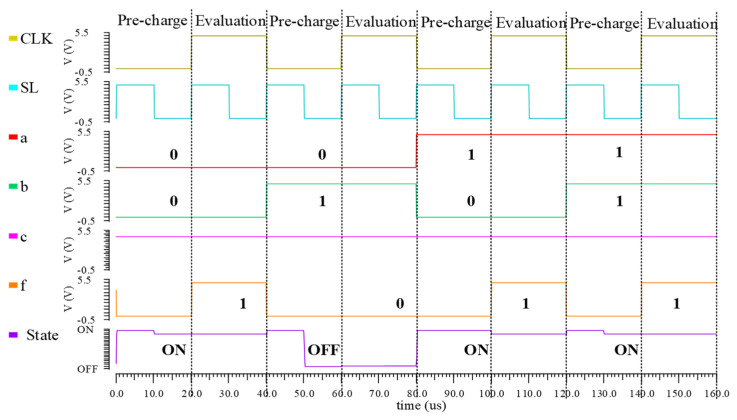
Simulation of the IMP logic gate with the complementary input *c* = 1.

When the complementary input is constant, the unit circuit performs a constant logic operation. Figure 7a describes that when the complementary input *c* = 1, the IMP logic gate will be performed between two independent inputs *a* and *b*. In the pre-charging stage, the state of the FeFET will be set to the ON state by the constant *c* = 1, and the state will be switched to the OFF only when the gate-source voltage difference is negative (corresponding to *a* = 0, *b* = 1). Since the OFF state will output as a low voltage *f* = 0 in the evaluation stage, the logical function to be implemented can be clearly determined based on the output voltage. Similarly, when the complementary input *c* = 0, the state of the FeFET will first be set to the OFF state, and the state will only switch to ON when the independent inputs *a* = 1 and *b* = 0. At this time, NIMP logic $f=a•\bar{b}$ will be achieved. The simulation, presented in Figure 8, demonstrates the constant logic operation IMP.

When the complementary input is a variable, the unit circuit performs a variable logic operation. Figure 7b shows the built-in majority logic gate of three variables *a*, *b*, and *c*. A conversion table as shown in Table 1 can be established based on the inputs and the output.

It can be clearly seen from the conversion table that the output signal can be determined based on the three inputs, corresponding to the non-volatile state one by one. The mathematical description of the built-in majority logic gate is:

(3) $$f=a•\bar{b}+a•c+\bar{b}•c=\mathrm{BM}(a,b,c).$$

**Figure 9:**
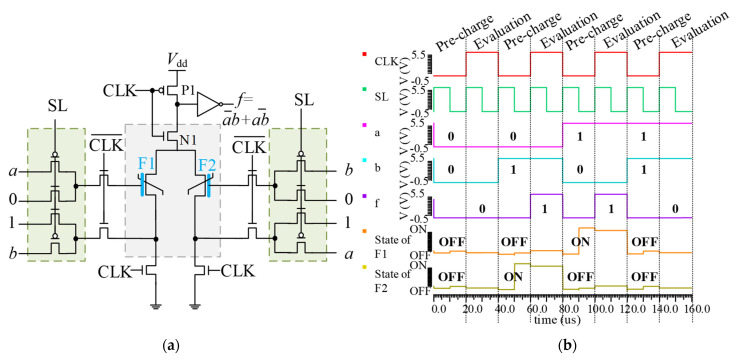
Schematic of the XOR logic gate: (**a**) the FeFET-based XOR; (**b**) simulation of the FeFET-based XOR.

When multiple FeFETs are connected in series or parallel in the logic network, more complex operations can be achieved. Figure 9a depicts an XOR logic gate implemented by two FeFETs in parallel while the complementary input *c* = 0, and Figure 9b confirms the XOR behavior.

Arbitrary functions can be synthesized using logic gates built by FeFET-based unit circuits. The uniformity of input and output variables facilitates a large-scale circuit design using existing logic synthesis tools. At the same time, in situ storage of operation results allows us to avoid reloading these data each time the system is powered, and ensures energy efficiency. The advantage of this design is to reduce the number of data transfer from the main system memory in applications such as IoT.

### 3.3. Full Adder Based on Unit Circuit

Whether performing simple arithmetic operations or complex data processing tasks, addition is one of the most basic operations. There are several FA designs based on NV devices, which only use NV devices to store operands. The operation results, especially the carry output, cannot be directly backed up, resulting in frequent data access and even data loss in power-off environments.

By considering that the FeFET is a logical element and not just a memory element, it is possible to design a more efficient FA by performing logic operations and in situ storage based on the proposed unit circuit. For a 1-bit FA, the mathematic equation can be expressed as:

(4) $$\left\{\begin{array}{l} Cout=ac_{in}+bc_{in}+ab=\mathrm{BM}(a,\bar{b},c_{in})\\ S=a⊕b⊕c_{in}=\bar{Cout}(a+b+c_{in})+abc_{in} \end{array}\right.,$$

where *a* and *b* are the addends, $c_{in}$ is the input carry, *S* and *Cout* are the output sum and output carry, respectively.

**Figure 10:**
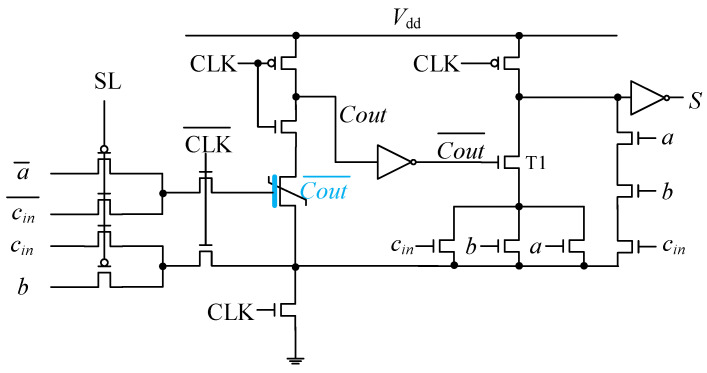
Architecture of a 1-bit FA with FeFET-based unit circuit to obtain nonvolatile carry output.

According to (4), *Cout* can be obtained through a BM gate, so we use the unit circuit to replace the carry output part constructed by NMOS gates in traditional DL FA circuits. In order to ensure that the output of $\bar{Cout}$ can normally control the NMOS that constitutes the output sum during the evaluation phase, FeFET is used to calculate and store the operation result of $\bar{Cout}$. The overall circuit is shown in Figure 10.

By adjusting the inputs of unit circuit, the FeFET can perform the $\bar{Cout}$ operation in the pre-charge phase of DL and store result in a non-volatile state. In the evaluation phase, the result is read out as a voltage and used to drive the NMOS T1, which is interconnected with other NMOS to realize the output sum *S*.

**Figure 11:**
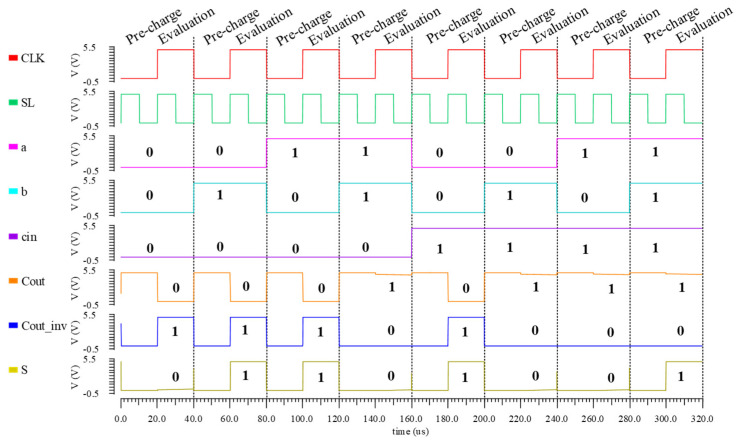
Simulation of the 1-bit proposed CiM FA based on the FeFET unit circuit.

All combinations of inputs have been tested, and the simulation waveform is shown in Figure 11. Compared with the output sum, the output carry is obtained by the FeFET-based unit circuit and stored in situ. In multi-bit serial FA, there is no need to read the result from memory, which reduces the handling of intermediate data and ensures efficient data recovery. On the contrary, the output sum designed only by NMOS requires additional memories to realize data backup.

## 4. Evaluation and Discussion

In this section, we present the performance study of our CiM FA circuit based on FeFET unit circuit and compare it with designs based on CMOS and FeFET technologies. We also provide a discussion of unit circuit application scenarios to explore the design directions of more efficient CiM circuits.

### 4.1. Evaluation of CiM FAs

The traditional CMOS-based DL FA, FeFET-based DL CiM FA design [14], and FeFET-based array FA design [31] are studied, and we simulate and compare them with the proposed CiM FA based on the FeFET unit circuit design under the same process environment using Cadence Virtuoso. The metrics we study include transistor count, delay, energy consumption, and operation cycle. The simulation results of the FeFET-based design are based on the FeFET device model discussed in Section 2.2. Table 2 summarizes the data for different FA designs.

Considering that CMOS DL FA is the most classic design, and the FeFET-based DL FA replaces the NMOS corresponding to some input variables (such as carry input *cin* or addend *b*) with FeFET, although the structure is the same, the control transistor used for data writing increases the number of transistors. The CiM FA based on the FeFET unit circuit realizes the complete carry output module function in one FeFET element. Although the number of transistors in the logic network has been greatly reduced compared with existing research, the realization of the carry function depends on the orderly transmission of logic inputs, which further increases the number of control transistors. However, the FeFET-based unit circuit can store the calculation results of the intermediate process in situ, which is more conducive to data recovery. On the contrary, in CMOS DL FA or even FeFET DL FA, since the calculation results (such as carry output *Cout*) are not backed up in time, they rely on additional memories (consuming 8 MOS). Therefore, when considering the function of data backup, the FeFET-based unit circuit has a leading advantage in saving hardware resources.

In terms of delay, both FeFET DL FA and FeFET-based unit circuit switch the FeFET state in the DL pre-charge phase, so the delay in the evaluation phase is determined by the flip speed of the clock. In terms of energy consumption, the CMOS DL FA and the FeFET DL FA have similar topologies. The FeFET-based unit circuit completely replaces the carry structure, while the sum output adopts the traditional structure. Their similar topology makes the three designs have similar delays and total power.

The design of FeFET-based array FA adopts a completely different memory array design idea from DL. Each array unit consists of a FeFET and an NMOS to realize the minimum function of FA. The calculation results of multiple array cells are read out in parallel in the form of current. In the FeFET-based array, the state initialization and conversion of voltage and current signals rely on the complex control of the peripheral circuit, but [31] does not introduce the peripheral circuit design and energy consumption data in detail. Here, we focus on comparing the transistor count and operation cycles of the array design and the unit circuit design.

Regarding the transistor count, the FeFET-based array cell can only realize two-input operations, which shows inferior logical expression ability to the three-input FeFET-based unit circuit. Therefore, at least 10 array cells are required to realize the FA function. In addition, the operation results are read out in the form of current, which means that there is no non-volatility and data backup is still required in the peripheral circuit. In terms of the operation cycle, since the array cell needs to write input data in advance, the overall operation cycle needs to include an additional “initialization” process. The DL-based unit circuit unifies the input and output forms. The Vin–Vout operation mode allows the unit circuits to be arbitrarily interconnected and no longer requires additional operation steps and peripheral conversion circuits. Therefore, there is a significant improvement in circuit cascading and computing efficiency.

The above analysis shows that the proposed FeFET-based unit circuit unifies the input forms, realizes the operation in a single device, and the input and output voltage range of the operation is constant, ensuring the cascading and scalability of the operation. The basic logic gates designed by FeFET-based unit circuit can be added to the traditional circuit design, not only without obvious loss of energy consumption and delay, but also reducing the additional storage units due to the in situ storage of the operation results. The FeFET-based unit circuit design can fully reflect the advantages of CiM architecture and is more conducive to the computing reliability in an unstable power supply environment.

### 4.2. Discussion on Application Opportunities

The proposed FeFET-based unit circuit can be embedded in traditional circuit design, and the design of direct backup of operation results is suitable for systems supplied by energy harvesting technology. The logic gates designed based on the FeFET unit circuit can expand the NV logic unit library.

If FeFET-based unit circuits are widely used in integrated circuit design, a large number of intermediate calculation results can be stored inside the computing system. The basic logic gates designed through unit circuits can be integrated into logic synthesis tools to create a synthesis flow of CiM units, and to jointly explore the optimization space of energy consumption and delay from the physical layer to the logical layer.

Since the proposed FeFET-based unit circuit can change the logic function by adjusting the signal transmission timing of the input network, it has reconfigurable capabilities. once the circuit is configured, there is no need to reconfigure it each time the circuit is powered, leading to increase the energy efficiency of the system. This concept can be applied to other applications such as achieving hardware encryption.

## 5. Conclusions

This paper outlines the CiM design concept of FeFET-based unit circuit. The proposed unit circuit overcomes the limitations of the conversion circuit, unifies the input forms, and establishes the Vin–Vout operation relationship with the FeFET switch state as a bridge. Non-volatile logic gates based on the unit circuit are provided and the proposed built-in majority gate is used to design the CiM FA. All circuits are successfully tested under the same conditions. The simulation results show that FeFET-based unit circuit can perform logic operation and data storage at the same time, which is beneficial for more efficient CiM circuit designs.

## Figures and Tables

**Table 1:** The transition table of the FeFET-based built-in majority logic gate.

Inputs				Output
*c*	*a*	*b*	State	*f*
0	0	0	OFF	0
0	0	1	OFF	0
0	1	0	ON	1
0	1	1	OFF	0
1	0	0	ON	1
1	0	1	OFF	0
1	1	0	ON	1
1	1	1	ON	1

**Table 2:** Performance of FAs.

Design	Transistor Count	Transistor Count (with Cout Recovery)	Delay (ps)	Energy (μW)	Operation Cycle
CMOS DL	19 MOS	27 MOS	119.4	4.95	1
FeFET DL	18 MOS + 3 FeFET	26 MOS + 3 FeFET	126.5	5.38	1
FeFET Array	10MOS + 10FeFET	18MOS + 10FeFET	—	—	2
FeFET-based Unit Circuit	21 MOS + 1 FeFET	21 MOS + 1 FeFET	123.7	5.69	1

## Data Availability

Data is contained within the article.
